# Gold nanoflower‐based surface‐enhanced Raman probes for pH mapping of tumor cell microenviroment

**DOI:** 10.1111/cpr.12618

**Published:** 2019-04-29

**Authors:** Mo Xie, Fan Li, Peilin Gu, Fei Wang, Zhibei Qu, Jiang Li, Lihua Wang, Xiaolei Zuo, Xueli Zhang, Jianlei Shen

**Affiliations:** ^1^ Shanghai Institute of Applied Physics Chinese Academy of Sciences Shanghai China; ^2^ University of Chinese Academy of Sciences Beijing China; ^3^ Institute of Molecular Medicine, Renji Hospital, School of Medicine Shanghai Jiao Tong University Shanghai China; ^4^ Joint Research Center for Precision Medicine Shanghai Jiao Tong University & Affiliated Sixth People's Hospital South Campus, Southern Medical University Affiliated Fengxian Hospital Shanghai China

## Abstract

**Objectives:**

Early diagnosis of tumour cells is critically important for cancer treatment. Given that the tumour environment is slightly acidic, the pH value of the cell environment can be used as a criterion for tumour diagnosis. However, mapping pH in the cell environment with high resolution, high sensitivity and accuracy remains challenging.

**Materials and Methods:**

Based on gold nanoflower as surface‐enhanced Raman scattering (SERS) substrate loading with p‐mercaptobenzoic acid (MPA) as pH‐responsive Raman reporter, a new SERS nanoprobe for pH mapping was developed.

**Results:**

This probe showed a characteristic Raman spectrum signal in response to the different pH in solutions or cells. The signal intensity is positively correlated to the pH value. Moreover, this probe is self‐correctable, which can help eliminate the influence of probe concentration on the accuracy of pH measuring.

**Conclusions:**

We demonstrate the pH mapping of cell environment using the probe, which can be used to distinguish normal cells and tumour cells. This method may provide a new imaging tool for early diagnosis of cancer.

## 1 INTRODUCTION

Cancer is a major killer that threatens the health of human life.^1^, ^2^ For advanced cancer, it is more difficult to remove tumour tissue by surgery for the metastasis and spread of tumour cells, and the recurrence rate is high. Based on this, early diagnosis is considered as the key factor for cancer treatment.^3^, ^4^, ^5^, ^6^, ^7^, ^8^, ^9^ At present, cancer diagnosis is mainly based on imaging methods,^10^, ^11^, ^12^ such as X‐ray computed tomography (CT),^13^, ^14^ magnetic resonance spectroscopy (MRS),^15^ magnetic resonance image (MRI),^16^, ^17^, ^18^ positron emission tomography (PET)^19^ and other technologies.^20^, ^21^ However, it is difficult to find the small tumour cell clusters and metastases with these techniques for low spatial resolution and low sensitivity.

Recent studies have indicated that the microenvironment of the tumour is weakly acidic, while the normal tissue is neutral/weakly alkaline.^22^, ^23^, ^24^ Therefore, early diagnosis of tumour could be achieved with monitoring the pH microenvironment of tissues.^25^, ^26^, ^27^, ^28^ For this point, a variety of fluorescent pH probes have been used for the development of tumour imaging methods.^29^, ^30^, ^31^ Fluorescence imaging methods possess higher resolution and sensitivity compared with other imaging ways.^32^, ^33^ However, there are some disadvantages for this kind of imaging. For example, the fluorescence molecules were poor in photostability and anti‐interference ability.^34^ In addition, the signal intensity of the pH‐responsive fluorescent probe is difficult to achieve quantitative analysis. Therefore, it is important to develop a pH imaging method that combines high sensitivity, stability and quantitative analysis.

Surface‐enhanced Raman spectroscopy (SERS) is a highly sensitive non‐destructive detection technology.^35^, ^36^ Based on this technology, detection sensitivity could be improved to reach 14 orders of magnitude higher than conventional Raman spectroscopy,^37^ which is comparable to fluorescence detection. The extremely short fluorescence lifetime of SERS reduces the photobleaching effect and the half‐peak width of the scattering peak compared to the fluorescent method.^38^ At present, various metals (such as Au, Ag, Co, Ni) can be used as the base material of SERS.^39^, ^40^, ^41^, ^42^ Moreover, previous studies have shown that the SERS effect is related to the surface roughness of nanomaterials.^43^, ^44^ In this paper, gold nanoflowers (AuNPs) with spiny protrusions on the surface were used as the SERS substrate, and p‐mercaptobenzoic acid (MBA) working as the Raman reporter was modified on the surface of AuNFs. In addition, MBA has the advantages of simple structure, easy bonding with gold surface, sensitivity to pH and high photochemical stability.^45^, ^46^ Base on that, MBA‐functioned AuNFs SERS nanoprobes can respond to different pH conditions with the SERS technology. At the same time, based on the fingerprint effect of the Raman scattering signal, the self‐calibration of the signal can be achieved by using the Raman peak which is less sensitive to pH change as the reference. In this way, the influence of the probe concentration is eliminated. Moreover, we propose that the SERS pH nanoprobes can be used to detect the acidity and alkalinity of the cell microenvironment, which would improve the development of early diagnosis methods of tumours.

## 2 MATERIALS AND METHODS

### 2.1 Experimental materials and apparatus

All the chemicals and reagents were purchased from Sigma‐Aldrich without any further purification unless otherwise stated. HPLC purified ssDNA (5′‐SH‐polyA30‐3′, 5′‐SH‐AAAAA AAAAA AAAAA AAAAA AAAAA AAAAA‐3′) was purchased from TaKaRa Biotechnology Co. Ltd. The AuNPs (15 nm) were purchased from BBI Co. Ltd. Nano pure water (>18 MΩ, MilliQ) was used in all experiments.

Transmission electron microscopy (TEM) images were taken with a Tecnai instrument (FEI). Raman measurement was performed on the XPLORA (Horiba) Raman microscope system. The dark‐field measurements were carried out on an inverted microscope (Olympus IX71). UV‐vis absorption obtained with a UV‐3100 (Hitachi) UV‐vis spectrophotometer.

### 2.2 Preparation of gold nanoflowers (AuNFs) SERS pH nanoprobes

#### 2.2.1 Preparation of DNA‐modified AuNPs

For the preparation of DNA‐modified AuNPs, the ssDNA (5′‐SH‐polyA30‐3′) was mixed with 15‐nm AuNPs solution of 12 nmol/L with 300:1 concentration ratio. After overnight incubation, the mixtures were adjusted to obtain a final phosphate concentration of 10 mmol/L (pH 7.4) with 100 mmol/L phosphate buffer. Then, the mixtures were adjusted to 0.2 mol/L NaCl with adding 2 mol/L NaCl every 30 minutes and then were allowed to shake overnight. Next, the resulting solution was washed three times in 10 mmol/L PB solution (pH 7.4) by centrifugation (15294 g, 20 minutes, 4°C). Finally, the precipitate was re‐dispersed in a solution (0.1 mol/L NaCl, 10 mmol/L PB, pH 7.4) to the final concentration of 10 nmol/L for next step.

#### 2.2.2 Preparation of AuNFs

For the preparation of gold nanoflower, 10 µL of the above colloid solution was mixed with 135 µL of 100 mmol/L PB solution, 5 µL of PVP (1%, w/v) solution and 50 µL hydroxylamine hydrochloride (3.5 mg/mL) solution. Then, 15 µL of chloroauric acid solutions (2‰, w/v) was added into above mixture under violent vibration and keep vibrating for l minute. After centrifuged at 12 000 rpm for 20 minutes, the precipitate of AuNFs was re‐dispersed in a H_2_O to the final concentration of 2 nmol/L. The AuNFs were characterized with a TEM and a UV‐vis spectrophotometer. The SERS enhancement factor (EF) calculation was followed according to previous work.

#### 2.2.3 Preparation of AuNFs SERS pH Nanoprobes

For synthesis SERS pH nanoprobes, 2 µL of Raman reporter MBA (0.1 mol/L) solution was added to the 100 µL gold nanoflower solution (2 nmol/L). The mixture was incubated at room temperature overnight, and then, the excessive Raman reporters were removed by washing it three times.

### 2.3 SERS Detection with different pH condition

SERS measurements were taken using above listed XPLORA Raman microscope system. Raman scattering was collected at a spectral resolution of 4 cm^−1^ with the range of 600‐2200 cm^−1^. The samples were prepared by dropping 5 µL of AuNFs SERS solution with different pH (1, 2, 3, 4, 5, 6, 7, 8, 9, 10, 11 and 12) in different condition (H_2_O and cell culture medium) on the silicon base.

### 2.4 Cellular imaging under Dark‐Field Microscope (DFM)

HEK 293 and Hela cells were incubated, respectively, with AuNFs SERS pH nanoprobes for 4 hours. They were then washed for three times with phosphate‐buffered saline (PBS). After that, cells were fixed by 4% formaldehyde and imaged under a dark‐field microscopy.

### 2.5 Cellular imaging under Raman confocal microscope

HEK 293 and Hela cells were incubated, respectively, with AuNFs SERS pH nanoprobes for 4 hours. The final concentrations of them were 1 nmol/L. After washing with phosphate‐buffered saline (PBS) for three times, cells were fixed by 2.5% glutaraldehyde and imaged under a Raman confocal microscope listed above.

## 3 RESULTS

### 3.1 Synthesis and characterization of AuNFs SERS pH nanoprobes

**Figure 1:**
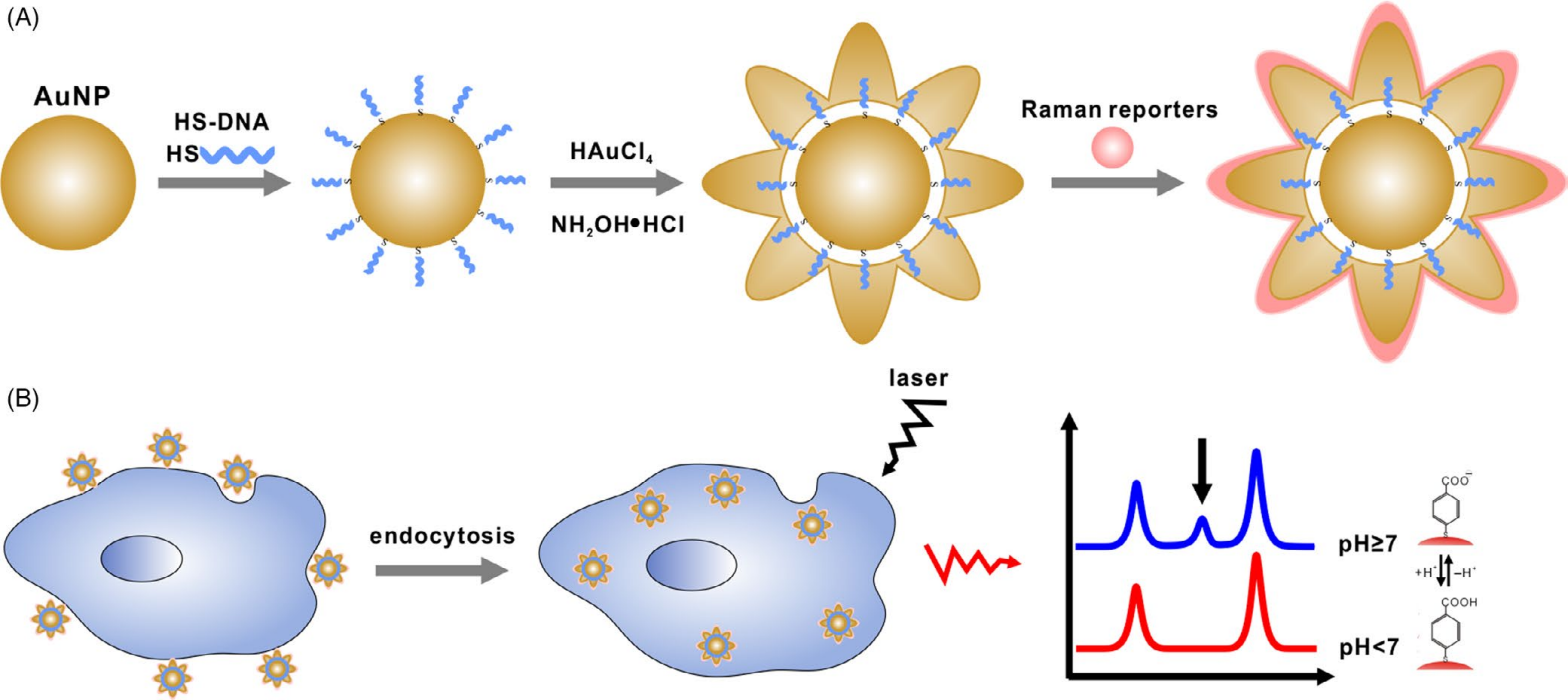
Schematic demonstration of the surface‐enhanced Raman scattering (SERS) pH nanoprobe‐based cell imaging. A, Steps to prepare AuNFs pH nanoprobe; B, SERS imaging of cells with different pH

As depicted in Figure 1A, the AuNFs SERS pH nanoprobes were synthesized based on functionalization of AuNFs with pH reporters of p‐mercaptobenzoic acid (MBA) molecules. In this method, SH‐polyA‐modified AuNPs were used as seeds to synthesis a gold nanoflower, with subsequent addition of polymer (PVP), reductant (NH_2_OH·HCl) and gold precursor (chloroauric acid solution, HAuCl_4_). Then, small Raman reporter molecules, MBA, were loaded onto the surface of the AuNFs via Au‐S bonds, and its SERS signal could be greatly enhanced based on the spiny morphology of AuNFs. For another, previous studies have reported that MBA Raman spectra would show a new resonance peak at 1428 cm^−1^ for the deprotonation of ‐COOH under neutral or alkaline pH environment.^45^ Besides, the peak intensity would increase with pH increasing. More importantly, the SERS pH nanoprobe could be taken up via endocytosis.^47^ Based on that, MBA‐functioned AuNFs could be used as a sensitive SERS pH nanoprobe, which further used to pH mapping of tumour cell microenvironment as shown in Figure 1B.

**Figure 2:**
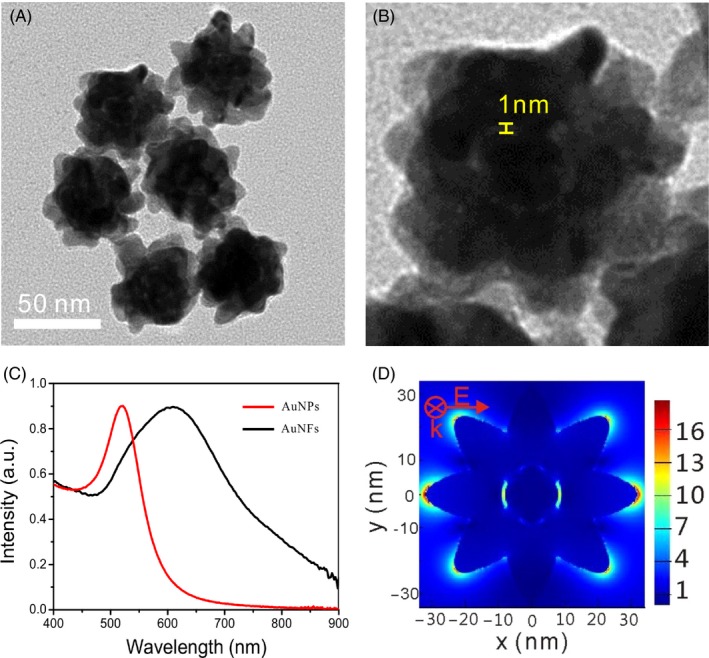
Characterization of AuNFs. A, Transmission electron microscopy (TEM) image of AuNFs; B, TEM image of single AuNF and its interior nanogap; C, UV‐vis spectrums of AuNPs and AuNFs; D, finite‐difference time‐domain simulation of electromagnetic field distribution of AuNFs

Following the above‐described synthesis way, uniform AuNFs with almost 50 nm size were obtained as shown in Figure 2A. As reported in previous literature of our group, the AuNFs have uniform surface spines and interior nanogaps (yellow segment) as shown in the TEM images in Figure 2B, and the gap size was almost 1 nm. Former work has indicated that polyA used in the synthetic process played a key role with blocking the direct deposition of gold onto the gold seed surface in forming the nanogap‐containing particles.^48^ With the formation of spines and nanoshell, the absorbance peak of nanoparticles red shifts from 520 to 608 nm as shown in Figure 2C.^49^, ^50^ The UV‐vis spectrum of AuNFs in our research was consistent with results published previously. The near‐field electromagnetic field distribution of the nanostructure was calculated using the finite‐difference time‐domain (FDTD) method with the acquired structural parameters (Figure 2D).^51^, ^52^ The FDTD simulation confirmed that the incident electric field would form a localized electric field (hot spot) in the tip of the surface spines and the nanocavity. The modified MBA Raman signal on the spines would be greatly enhanced due to the surface‐enhanced Raman effect.

### 3.2 Analytical performance evaluation of the SERS pH nanoprobe

**Figure 3:**
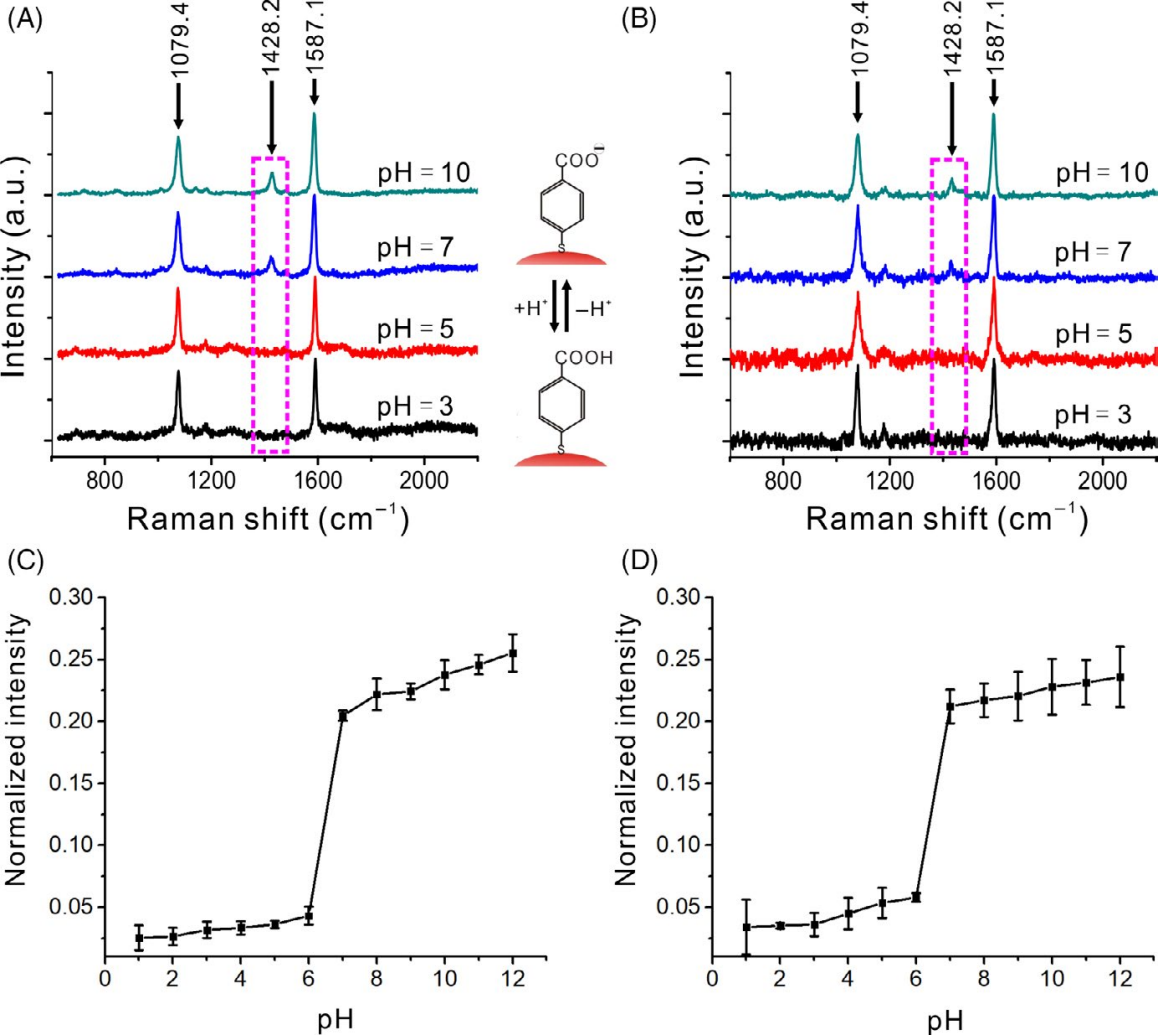
Raman spectra analysis of nanoprobes under several pH conditions (pH = 3, 5, 7 and 10) in H_2_O solution (A) and cell culture medium (B). pH normalization curves of pH sensitive peak (1428.2 cm^−1^) in H_2_O solution (C) and cell culture medium (D)

To bring these SERS pH nanoprobes into practical applications, the pH nanoprobe was evaluated in standard H_2_O solution with different pH situations. As shown in Figure 3A, AuNFs SERS pH nanoprobes showed strong Raman signals at different pH conditions. There were two absorption peaks at 1079.4 and 1587.1 cm^−1^ correspond to the vibrational peaks of ν8a and ν12 benzene rings, respectively. In the acidic environment, the carboxyl group exists in protonated form. But in the neutral and alkaline environment, the carboxyl group was deprotonated with COO^−^ form, and the Raman spectrum showed a weak absorption peak near 1428.2 cm^−1^, which corresponded to COO^−^ groups. At the same time, the intensity of the absorption peak near 1428.2 cm^−1^ was positively correlated with pH (Figure 3C). This result indicates that the prepared SERS pH nanoprobe could indicate the pH change of the solution well. We also noticed that there was a sudden change of Raman intensity around pH 7 which be the isoelectric point of SERS pH nanoprobe.

Encouraged by the above investigations, the SERS pH nanoprobes were also evaluated in cell culture medium. As shown in Figure 3B,D, the change in SERS signal in the cell culture medium was similar to the change in the aqueous phase. It is indicated that the stability of the nanoprobe was not affected in the cell culture medium environment, and it still possesses good response ability to pH.

### 3.3 Cell uptake efficiency and biocompatibility evaluation based on Dark‐Field Image (DFI)

**Figure 4:**
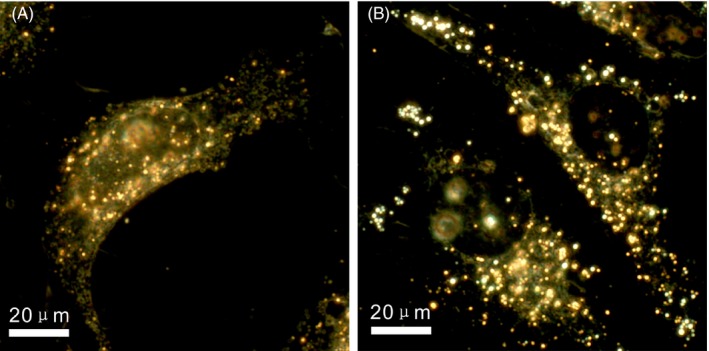
Dark‐field image of HEK 293 cell (A) and Hela cell (B) after treated with nanoprobes for 4 h

The cell uptake efficiency of SERS pH nanoprobes was evaluated with dark‐field microscopy. The nanoprobes were incubated with normal human cells (HEK 293) and tumour cells (Hela) for 4 hours, and then, dark‐field imaging was performed. As shown in Figure 4, the nanoprobes showed high cell uptake efficiency for normal cells and tumour cells. At the same time, both cell morphologies were normal, which indicated good biocompatibility of the SERS pH nanoprobe.

### 3.4 pH mapping for cell analysis based on SERS pH nanoprobes

**Figure 5:**
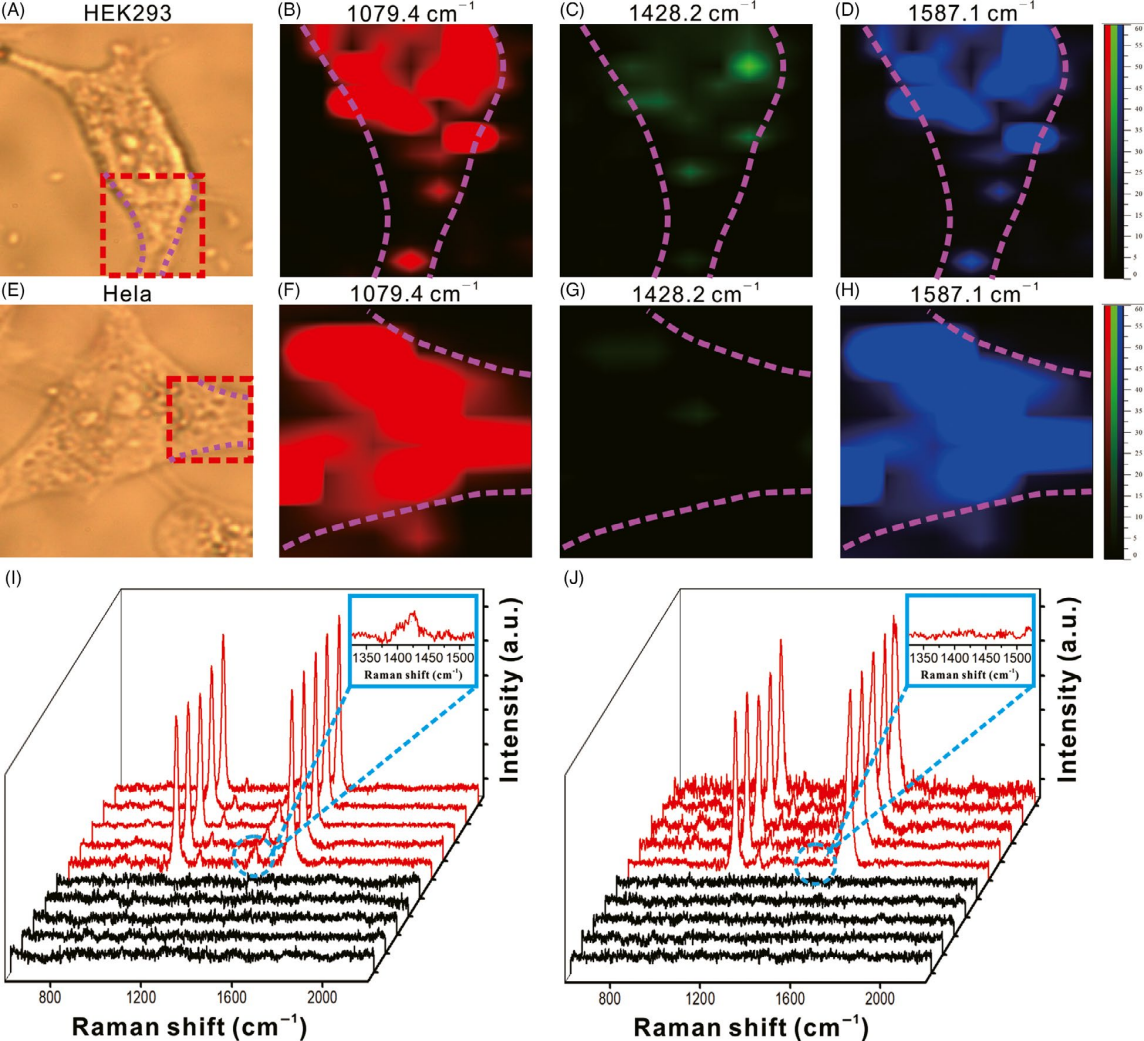
Identification of tumour cell via Raman imaging. (A) and (E) are the optical image of HEK 293 cell and Hela cell. (B‐D) Raman mapping image of the nanoprobes distribution inside HEK 293 cell and Hela cell under different peak mode (1079.2, 1428.2 and 1587.1 cm^−1^). (I) Surface‐enhanced Raman scattering spectra collected from intracellular (red) and extracellular (black) area of HEK 293 cell and Hela cell (J)

To validate the practicability of SERS pH nanoprobes, two different kinds of cells were selected for Raman imaging, which were normal HEK 293 cells and tumour Hela cells. The performance of SERS pH nanoprobes for intercellular pH mapping was investigated by using a streamline Raman mapping system. After incubated nanoprobes with the cells, the laser confocal Raman imaging was performed in local areas of normal HEK 293 cells and tumour Hela cells, respectively (Figure 5). As seen from the Raman mapping maps (Figure 5C,G), normal cells showed clearer signals at 1420 cm^−1^ compared with tumour cells. The Raman spectroscopy showed the same trend as shown in Figure 5I,J. Compared with tumour cells, normal cells showed a new weak absorption peak corresponding to COO^−^ at 1428.2 cm^−1^, indicating that the MBA molecules deprotonated to produce COO^−^ in normal cells, but was in the form of COOH in tumour cells. The results were also consistent with the actual situation reported in the literature, which the normal cell environment was neutral or alkaline, and the tumour cell environment was acidic. Raman spectroscopy was also performed at random points of intracellular and extracellular (Figure 5I,J). It could be seen that the Raman signal was only detected in intercellular, which proved cell uptake status of nanoprobes from another side. The result of HEK293 and Hela cells pH mapping indicated that AuNFs SERS pH nanoprobes could be used for cell analysis with differentiating normal cells and cancer cells.

## 4 DISCUSSION

We developed a SERS pH nanoprobe based on MBA‐modified AuNFs. Based on different status of the –COOH group of MBA molecules under different pH condition, the SERS pH nanoprobe showed pH‐dependent Raman signals. More importantly, the spines of AuNFs surfaces could form a high‐efficiency electric field, called hot spot, and realized these Raman signals enhancement. Therefore, this SERS pH nanoprobe achieved sensitive response to pH and could differentiate normal cells and tumour cells with pH Raman mapping. The probe had the advantages of high signal intensity, high signal‐to‐noise ratio, good biocompatibility and strong photostability. This technology provided a new imaging method for early diagnosis of cell‐level tumours.

## CONFLICT OF INTEREST

The authors declare that they have no conflict of interest.
